# Impact of Educational Films on Antibiotic Prescription among Physicians: A Web-Based Survey in Japan

**DOI:** 10.3390/antibiotics13080724

**Published:** 2024-08-01

**Authors:** Kosaku Komiya, Ryohei Kudoh, Norihito Kaku, Yuichiro Shindo, Tatsuya Hayashi, Kei Kasahara, Tomohiro Oishi, Naruhiko Ishiwada, Makoto Ito, Hiroshi Yotsuyanagi, Naoki Hasegawa, Kazuhiro Tateda, Muneki Hotomi, Katsunori Yanagihara

**Affiliations:** 1 Respiratory Medicine and Infectious Diseases, Oita University Faculty of Medicine, 1-1 Idaigaoka, Hasama-machi, Yufu, Oita 879-5593, Japan; 2 Department of Laboratory Medicine, Nagasaki University Hospital, 1-7-1 Sakamoto, Nagasaki 852-8501, Japan; 3 Department of Respiratory Medicine, Nagoya University Graduate School of Medicine, 65 Tsurumai-cho, Showa-ku, Nagoya 466-8550, Japan; 4 Department of Otolaryngology-Head and Neck Surgery, Asahikawa Medical University, Midorigaoka Higashi 2-1-1-1, Asahikawa 078-8510, Japan; 5 Center for Infectious Diseases, Nara Medical University, 840 Shijo-cho, Kashihara, Nara 634-8521, Japan; 6 Department of Clinical Infectious Diseases, Kawasaki Medical School, 577 Matsuyama, Kurashiki 701-0192, Japan; 7 Department of Infectious Diseases, Medical Mycology Research Center, Chiba University, 1-8-1 Inohana, Chuo-ku, Chiba 260-8673, Japan; ishiwada@faculty.chiba-u.jp; 8 Department of Otolaryngology and Head and Neck Surgery, Jichi Medical University, 3311-1 Yakushiji, Shimotsuke-shi, Tochigi 329-0498, Japan; 9 Department of Infectious Diseases and Applied Immunology, IMSUT Hospital, Institute of Medical Science, University of Tokyo, 4-6-1 Shirokanedai, Minato-ku, Tokyo 108-8639, Japan; 10 Department of Infectious Diseases, Keio University School of Medicine, 35 Shinano-machi, Shinjuku-ku, Tokyo 160-8582, Japan; 11 Department of Microbiology and Infectious Disease, Toho University School of Medicine, 5-21-16 Omori-nishi, Ota-ku, Tokyo 143-8540, Japan; 12 Department of Otorhinolaryngology-Head and Neck Surgery, Wakayama Medical University, 811-1 Kimiidera, Wakayama 641-8509, Japan

**Keywords:** antibiotics, respiratory tract infection, prescription, education, drug-resistant

## Abstract

Although antibiotics are most frequently prescribed for respiratory tract infections, effective interventions for their proper use by physicians have not been fully established. We assessed the impact of educational films on the rates of antibiotic prescriptions for respiratory tract infections using fictitious scenarios. In this nationwide web-based survey prospective study, a total of 1100 physicians were included. The physicians were required to view educational short films and determine the need for prescribing antibiotics in 10 fictitious scenarios involving adults diagnosed with different acute respiratory tract infectious diseases. The antibiotic prescription rates for each scenario were compared before and after viewing the educational short film. The rates of antibiotic prescription significantly decreased after viewing the educational film, especially in cases with a narrowly defined common cold (from 51% to 15%), mild pharyngolaryngitis (from 71% to 25%), and acute bronchitis without chronic respiratory underlying diseases (from 63% to 23%). Alternatively, a slight decrease in rates was observed in cases with moderate or severe rhinosinusitis (from 94% to 79%), moderate or severe acute pharyngitis (from 88% to 69%), and acute bronchitis with chronic lung disease (from 70% to 58%), for which antibiotics are recommended. Educational short films may encourage the proper use of antibiotics for respiratory tract infections; however, the possibility of undertreatment in patients requiring antibiotics must be considered.

## 1. Introduction

Antimicrobial resistance (AMR) is a growing public health threat worldwide. The World Health Organization adopted a global action plan for AMR in 2015 to optimize antibiotic use because inadequate regulations can increase the injudicious intake of antibiotics [1]. Notably, the isolation rates of antibiotic-resistant bacteria and the extent of antibiotic usage are positively correlated [2,3].

According to antibiotic prescription data linked to diagnosis in Japan, more than 60% of antibiotics were used for acute respiratory tract infection (RTI) [4]. Similarly, antibiotics were prescribed most frequently for acute respiratory conditions (41–44%) in the ambulatory care setting in the USA [5,6]. In another study using primary care data in the UK, 46% of antibiotics were prescribed for acute respiratory conditions [7]. These studies revealed that viral RTI was the most frequent infection associated with antibiotic prescription. A systematic review analyzing the reasons for antibiotic prescriptions for RTIs indicated that high fever, white lesions of the laryngopharynx, purulent sputum, and the patient’s desire were major factors related to the prescription of antibiotics [8]. However, these symptoms and findings are not critical for diagnosing bacterial infections.

The rate of improper antibiotic prescriptions may decrease if physicians are provided with accurate information via appropriate educational materials. The impact of educational interventions on antibiotic prescription has been assessed in different sites, and most studies indicate room for improvement in antibiotic prescription behaviors [9,10]. However, no interventional study with universal health insurance coverage has been conducted in Japan. This study aimed to assess the effects of an educational film on the intention to prescribe antibiotics and determine the factors susceptible or insusceptible to the educational intervention using fictional cases.

## 2. Results

### 2.1. Backgrounds of the Participants

A total of 1100 physicians (660 generalists, 220 pulmonologists, and 220 otorhinolaryngologists) with a median career length of >20 years for each expertise participated in this study. Pulmonologists tended to be more aware of the indications for antibiotic use for RTIs based on the Japanese Association of Infectious Diseases (JAID) recommendations; additionally, they attended the training sessions for infectious diseases more frequently and comprised a greater number of certified infectious disease specialists than generalists and otorhinolaryngologists. Generalists and otorhinolaryngologists were more commonly in charge of clinic or hospital management and examined children on a daily basis (Table 1).

### 2.2. Rates of Antibiotic Prescription before Viewing the Educational Film

**Figure 1:**
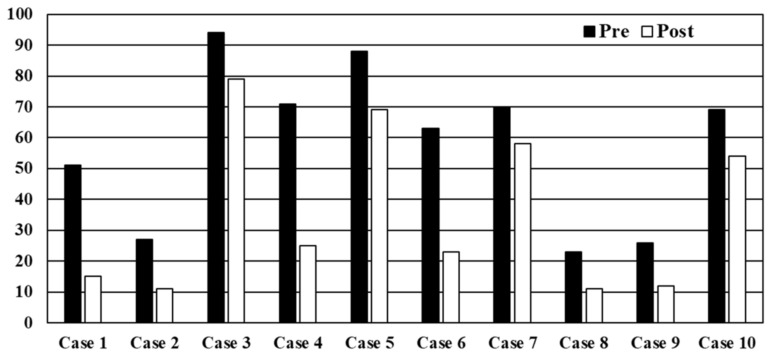
The rates of antibiotics prescription before and after viewing the educational film. *p* values were <0.001 in all cases.

**Figure 2:**
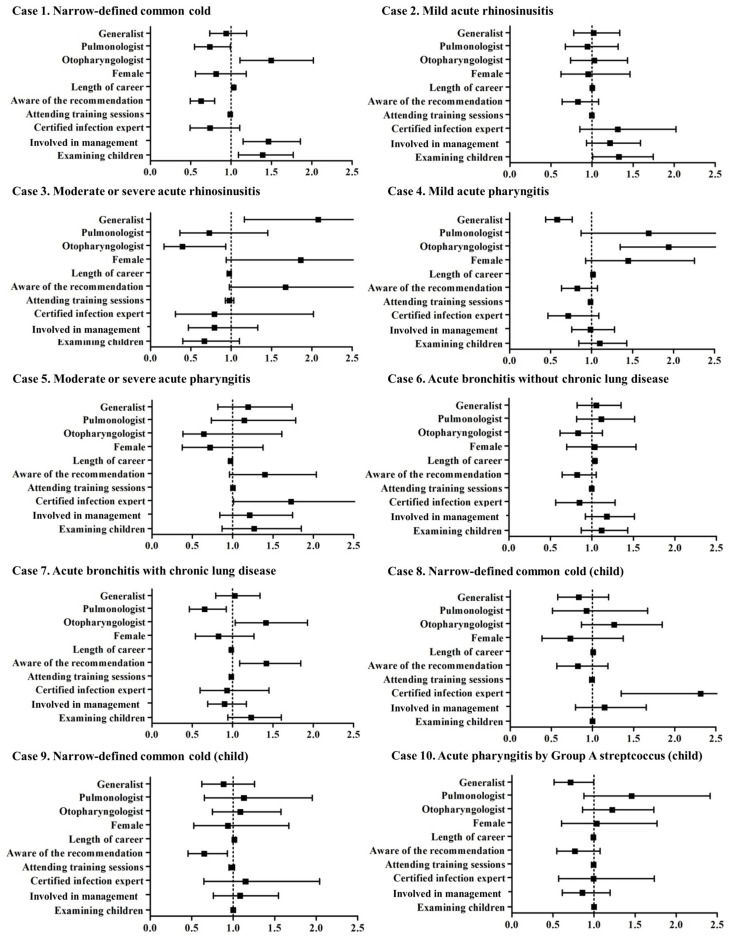
Odds ratios for improper use of antibiotics by physicians’ backgrounds before intervention. Improper use was defined as antibiotic and no-antibiotic prescriptions for cases not requiring (cases 1, 2, 4, 6, 8, and 9) and requiring antibiotic treatment (cases 3, 5, 7, and 10).

Antibiotics are not recommended for cases 1, 2, 4, 6, 8, and 9. However, the antibiotic prescription rates in case 1 (narrow-defined common cold), 4 (mild acute pharyngitis), and 6 (acute bronchitis without chronic lung disease) were >50%, whereas those in case 2 (mild acute rhinosinusitis), 8 (narrow-defined common cold in child), and 9 (narrow-defined common cold in child) were relatively low (Figure 1). The top five reasons for antibiotic prescription are summarized in Table S1. Redness of the pharynx, patient’s desire, fever in case 1 (narrow-defined common cold), white lesion of the laryngopharynx in case 4 (mild acute pharyngitis), and purulent sputum in case 6 (acute bronchitis without chronic lung disease) were the major reasons for antibiotic prescription. Otorhinolaryngologists, physicians participating in clinical or hospital management, and those who examined children every day were significantly associated with the improper use of antibiotics in case 1; likewise, otorhinolaryngologists were significantly associated with the improper use of antibiotics in case 4 (Figure 2). The detailed odds ratios are documented in Supplementary Table S2.

Antibiotics are recommended for cases 3, 5, 7, and 10, and approximately 70% to 90% of the physicians intended to prescribe antibiotics. As shown in Figure 2, generalists in case 3 (moderate or severe acute rhinosinusitis); certified infectious diseases specialists in case 5 (moderate or severe acute pharyngitis); and otorhinolaryngologists, as well as being aware of the recommendations for the proper use of antibiotics for RTIs by the JAID, in case 7 (acute bronchitis with chronic lung disease) were associated with refraining from prescribing antibiotics even for cases requiring antibiotics.

### 2.3. Effects of the Educational Film on the Proper Use of Antibiotics

After viewing the educational film, antibiotic prescription was significantly reduced in all cases (*p*-value < 0.001 in all scenarios). In particular, extreme reductions were significantly found in cases 1, 2, 4, 6, 8, and 9, which do not require antibiotic treatment. The reduction rates were 56% (from 51% to 15%) in case 1, 16% (from 27% to 11%) in case 2, 46% (from 71% to 25%) in case 4, 40% (from 63% to 23%) in case 6, 12% (from 23% to 11%) in case 8, and 13% (from 26% to 12%) in case 9. Reductions were also seen in cases 3 (15%; from 94% to 79%), 5 (19%; from 88% to 69%), 7 (12%; from 70% to 58%), and 10 (15%; from 69% to 54%), which required antibiotic treatment.

**Figure 3:**
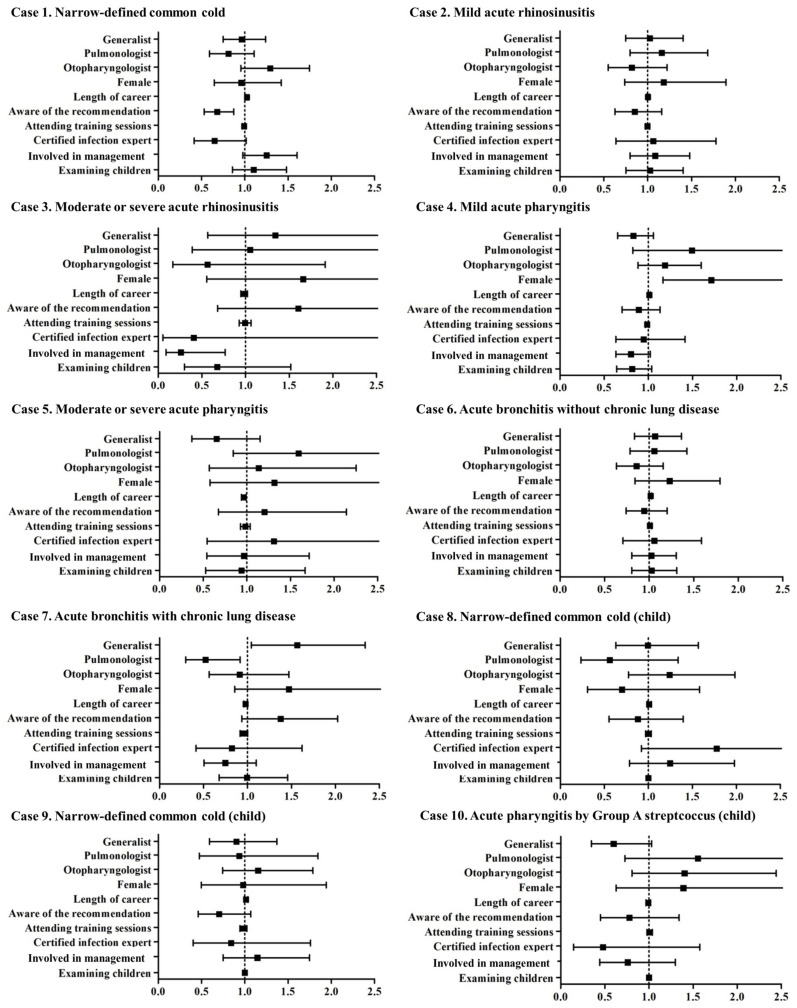
The positive effects of the educational film on the proper use of antibiotics by physicians’ backgrounds. A positive effect was defined as a change from an antibiotic prescription to a no-antibiotic prescription or vice versa in cases not requiring (cases 1, 2, 4, 6, 8, and 9) or requiring (cases 3, 5, 7, and 10) antibiotic treatment.

The positive effects of the educational film on the proper use of antibiotics, change from an antibiotic prescription to a no-antibiotics prescription or vice versa in cases not requiring (cases 1, 2, 4, 6, 8, and 9) or requiring antibiotic treatment (cases 3, 5, 7, and 10), were more commonly observed among otorhinolaryngologists and physicians participating in clinical or hospital management in cases 1, 4, and 7 (Figure 3). The detailed odds ratios are documented in Table S3. The post hoc power (1-beta error probability) analysis with an alpha error probability of 0.05 and a total sample size of 1100 showed values of 1.00, 1.00, 0.98, 1.00, 1.00, 1.00, 0.99, 1.00, 1.00, and 1.00 in cases 1, 2, 3, 4, 5, 6, 7, 8, 9, and 10, respectively.

## 3. Discussion

This study demonstrated the effects of educational intervention on the proper use of antibiotics for RTIs. The effects were more dramatically observed in conditions such as narrowly defined common cold, mild acute pharyngitis, and acute bronchitis without chronic lung disease in adults. In contrast, the rate of antibiotic prescriptions decreased slightly but significantly, even in cases that should be treated with antibiotics. The effects observed in this study must be discussed based on individual scenarios because the interventional effects varied depending on the physician’s background in the present study.

Case 1 is diagnosed as a narrowly defined common cold in an adult, and antibiotic treatment is not recommended. Otorhinolaryngologists, physicians involved in clinical or hospital management, and physicians who examined children daily were associated with antibiotic prescriptions. Based on the positive effect (i.e., change from an antibiotic prescription to a no-antibiotic prescription) of the educational film on physicians with these backgrounds, a lack of knowledge might be the primary reason for the improper prescription of antibiotics in these cases. Generalists were reported to prescribe several antibiotics for outpatients with any disease in the USA [11], possibly due to the lack of opportunities for patient follow-up [8]. In contrast, generalists working on RTIs in Japan appeared to have adequate knowledge about antibiotic use, especially in the case of a narrowly defined common cold.

Case 2 is diagnosed as mild acute rhinosinusitis in an adult, and antibiotics are not recommended. The antibiotic prescription rate was relatively low (27%) compared to other scenarios, but a significant reduction in antibiotic prescriptions was observed after viewing the educational film. Conversely, case 3 is diagnosed as moderate or severe acute rhinosinusitis, which requires antibiotic treatment. The prescription rate was high (94%) before the intervention. Although the educational films encouraged the prescription of antibiotics in this case, the rate of antibiotic prescription was slightly reduced after watching the movie. Generalists were associated with the improper use of antibiotics (refraining from antibiotics prescription in this case). Generalists might be less likely to prescribe antibiotics even for severe cases in Japan [8], and educational intervention, especially for generalists, should improve the treatment quality.

Case 4 is diagnosed as mild acute pharyngitis, which does not require antibiotic treatment. Nevertheless, the antibiotic prescription rate was high (71%) before viewing the film and decreased (25%) after the intervention. Otorhinolaryngologists are more likely to prescribe antibiotics improperly than generalists and pulmonologists, mainly due to the presence of a white lesion on the laryngopharynx. This feature does not directly indicate a bacterial infection and may frequently be observed in viral infections [12]. Alternatively, case 5 is diagnosed with moderate or severe acute pharyngitis requiring antibiotic treatment. Similar to moderate or severe rhinosinusitis (case 3), the antibiotic prescription rate was high (88%) initially but significantly decreased (69%) after viewing the film. Notably, certified infectious disease experts did not prescribe antibiotics for case 5, indicating that experts also need to be regularly educated with proper educational materials.

Cases 6 and 7 represented acute bronchitis without and with chronic lung disease, respectively. The indications for the antibiotic treatment for acute bronchitis should be determined depending on the presence of chronic pulmonary diseases [13]. The antibiotic prescription rate was high (63%) in acute bronchitis without chronic lung disease and with purulent sputum but dramatically reduced (23%) after the intervention. In contrast, physicians who answered “yes” to “aware of the recommendation for RTI created by the JAID” did not prescribe antibiotics for case 7 (acute bronchitis with chronic lung disease). This result may reflect the fact that some physicians do not correctly learn from the recommendations provided by official associations.

Cases 8 and 9 are diagnosed as narrowly defined common colds in children. The prescription rates were relatively low compared to the corresponding cases in adults. In 2018, the Ministry of Health, Labour and Welfare in Japan introduced an additional fee for physicians who did not prescribe antibiotics for viral infections, which may have affected the low antibiotic prescription rates in these two cases. Case 10 is diagnosed as acute pharyngitis caused by Group A streptococcus. The generalists refrained from antibiotic prescription for this case, similar to case 3 (moderate or severe rhinosinusitis in an adult), and the educational film did not successfully affect their decision. Thus, more effective approaches, other than viewing films, are needed to educate generalists who treat children on a daily basis.

Overall, viewing the educational films dramatically reduced the prescription of antibiotics in cases that do not require antibiotic treatment. Surprisingly, it slightly but significantly decreased the rate of antibiotic prescriptions in cases requiring antibiotic treatment. Presumably, the participants knew the concept of this survey and felt pressured to select the option of “treatment without antibiotics” for all cases. Although the educational intervention may reduce the improper use of antibiotics, it could lead to undertreatment for cases requiring antibiotics.

The strength of this study is the large-scale experimental research conducted using educational short films. However, it has several limitations. First, we set fictional scenarios and used questionnaires; the effects of the intervention might differ in the real world. For example, in cases with rhinosinusitis, the duration of symptoms was set as 3 (case 2) and 10 days (case 3) to avoid the gray zone of 5–10 days for the indication of antibiotic treatment. This study did not clarify the prescription behavior in such gray scenarios. Second, the health insurance system and medical access vary depending on the area or country. Japan has universal health coverage, and physicians may be prone to prescribing antibiotics depending on the patient’s request [14]. Finally, in this study, the physicians were required to respond to the questions immediately after viewing the film. Thus, the long-term impacts remain uncertain. Some studies conducted in other countries showed that long-term intervention could sustain the improved prescription behaviors [10]. Additional studies using sustainable approaches to maintain the effects are warranted.

In conclusion, the educational film beneficially impacted the intention to prescribe antibiotics, especially for the narrow-defined common cold, mild acute pharyngitis, and acute bronchitis without chronic lung disease. Otorhinolaryngologists, physicians involved in clinical or hospital management, and physicians who examine children daily benefit more from the intervention. Educational interventions using short films must be exploited to promote proper antibiotic prescriptions for RTIs among physicians.

## 4. Methods

### 4.1. Study Design and Participants

This prospective web-based interventional study employed an online network platform (PLAMED Inc., Tokyo, Japan), which is used to conduct various surveys on medical care for its members who work as physicians. A total of 660 generalists (including 330 working in clinics), 220 pulmonologists (including 39 working in clinics), and 220 otorhinolaryngologists (including 93 working in clinics), who were registered in the online network platform in advance, were targeted in this study. Information about the physicians’ backgrounds that could affect prescription behaviors—such as their career length, certified infection expertise status, and hospital management involvement—were collected. The sample size was determined based on the research fund because this was an exploratory survey targeting physicians with different expertise. A post hoc power analysis was conducted for each scenario.

This study was performed as part of a campaign for the proper use of antibiotics for RTIs in Japan and was conducted by the JAID. The committee consisted of four types of specialists—three otorhinolaryngologists (TH, MI, and MH), two pediatricians (TO and NI), two pulmonologists (KK and YS), and three infectious disease experts (NK, KK, and KY). This study was approved by the Institutional Ethics Committee of the Oita University Faculty of Medicine (approved no. 2509, 31 March 2023). Informed consent was obtained from all participants on the web screen before answering the questionnaires. All aspects of the study complied with the Declaration of Helsinki. This survey was financially supported by an Independent Medical Education Grant from Pfizer Japan Inc. (Tokyo, Japan)

### 4.2. Fictional Scenarios

The physicians were asked to determine whether antibiotics were indicated for the following fictitious scenarios and provide reasons for their decision. The reasons were selected from multiple choices (overlap allowed). Detailed information about the questionnaire is available in Table S4. The educational short film automatically and immediately started after completing the questionnaires; the participants had to answer the same questions for the same scenarios shortly after viewing the short film.

Ten fictional scenarios were set to cover seven RTIs in adults (narrowly defined common cold, mild acute rhinosinusitis, severe acute rhinosinusitis, mild acute pharyngolaryngitis, severe acute pharyngolaryngitis, acute bronchitis without chronic respiratory diseases, and acute bronchitis with chronic respiratory diseases) and three RTIs in children (narrowly defined common cold in a 2-year-old child, narrowly defined common cold in a 10-year-old child, and bacterial pharyngolaryngitis in a 5-year-old child; Table 2). A systematic review of the reasons for prescribing antibiotics for RTIs showed that the patients’ desire substantially affected their behavior [8]. Therefore, the patients in these scenarios were conditioned to want to be treated with antibiotics. The clinical presentations of each case are as follows:

Case 1. A male in his 50s visited the hospital with nasal discharge, a sore throat, a cough, and a fever (38 °C), which had been persistent for 3 days. Redness of the pharynx was observed, and the patient wanted to be treated with antibiotics.

Case 2. A male in his 50s presented with yellow nasal discharge and a fever (38 °C), which had been persistent for 3 days. He did not complain of a sore throat or a cough but wanted an antibiotic prescription.

Case 3. A male in his 50s presented with yellow nasal discharge and a fever (38 °C), which had been persistent for 10 days. He complained of severe facial pain without a sore throat and a cough and desired antibiotic treatment.

Case 4. A male in his 50s visited the hospital with a persistent sore throat, a cough, and a fever (37 °C), which had persisted for the past 5 days. He did not complain of nasal discharge. A white lesion was observed in the laryngopharynx, but the anterior cervical lymph nodes were not swollen. He desires antibiotic treatment.

Case 5. A male in his 50s visited the hospital with a persistent sore throat, loss of appetite, and a fever (37 °C), which had persisted for the past 5 days. He did not complain of nasal discharge or a cough. A white lesion was seen in the laryngopharynx, and the anterior cervical lymph nodes were swollen. He desired antibiotic treatment.

Case 6. A male in his 50s with no history of smoking visited the hospital with a cough, persistent purulent sputum, and a fever (37 °C), which had persisted for the past 5 days. He did not complain of nasal discharge or a sore throat. Abnormal lung involvement was not observed on the chest X-ray. He desired antibiotic treatment.

Case 7. A male in his 50s with a history of heavy smoking visited the hospital with a cough, persistent purulent sputum, and a fever (37 °C), which had persisted for the past 5 days. He did not complain of nasal discharge or a sore throat. Abnormal lung involvement was not observed on the chest X-ray. He expressed a desire for antibiotic treatment.

Case 8. A 2-year-old preschool child visited the hospital with nasal discharge, a persistent cough, which had been present for the past 3 days, and a fever (38 °C), which had been persistent for one day. The accompanying parents expressed a desire for antibiotic therapy.

Case 9. A 10-year-old child visited the hospital with nasal discharge and a persistent cough, which had been present for 3 days, and a fever (38 °C), which had been persistent for one day. The accompanying parents expressed a desire for antibiotic therapy.

Case 10. A 5-year-old child visited the hospital with a severe sore throat, a fever (38 °C), and a stomach ache, which had persisted for one day. There was no nasal discharge or cough. A white lesion was seen in the laryngopharynx, and the anterior cervical lymph nodes were swollen. The accompanying parents expressed a desire for antibiotic therapy.

### 4.3. Educational Movie

The educational film was created by the committee members of the JAID. The disease concept, classification of severity, and indications for antibiotics for RTIs (including narrowly defined common cold, acute rhinosinusitis, acute pharyngolaryngitis, and acute bronchitis) were followed according to international guidelines [15,16,17,18,19,20]. These guidelines do not routinely recommend blood tests with C-reactive protein or procalcitonin; they focus on the disease severity rather than the pathogen-based treatment, except for Group A streptococcal infections in laryngopharyngitis because point-of-care testing in the primary care setting is assumed. Therefore, these details were not included in the scenarios and educational films.

A summary of the educational movie was included after screening; if a patient had mixed symptoms, including nasal, laryngopharyngeal, and lower respiratory tract symptoms, “narrowly defined common cold” would be diagnosed. Most causative pathogens are viruses, and antibiotics are not required. If a patient predominantly complains of nasal symptoms, “acute rhinosinusitis” would be diagnosed. Even in the case of acute bacterial rhinosinusitis, mild cases may be resolved without antibiotics; only moderate or severe acute bacterial rhinosinusitis require antibiotics. If a patient predominantly complains of laryngopharyngeal symptoms, “acute laryngopharyngitis” would be diagnosed. Most cases are caused by viruses; however, acute laryngopharyngitis caused by Group A streptococcus indicates antibiotics. The Centor score is recommended for diagnosing Group A streptococcal infections. Finally, if a patient predominantly complains of lower respiratory tract symptoms such as a cough, “acute bronchitis” would be diagnosed. Most cases of acute bronchitis are caused by viruses, and antibiotic treatment is not needed. Bacterial bronchitis is suspected in patients with chronic respiratory diseases, and antibiotic treatment may be considered.

The short film (4 min and 58 s) is available at https://www.youtube.com/watch?app=desktop&v=yFsTKggL4IU (accessed on 17 November 2023) (English version) and https://www.youtube.com/watch?app=desktop&v=DPe4OJDFAyw (accessed on 17 November 2023) (Japanese version).

### 4.4. Statistical Analysis

Statistical analyses were performed using the Statistical Package for the Social Sciences version 22 (IBM Japan, Tokyo, Japan). For the two-tailed analyses, 95% confidence intervals were calculated. The rates of antibiotic prescriptions before and after viewing the film were compared using the Chi-square test. Associations between the physician’s background and the improper use of antibiotics or the positive effects of educational films were analyzed using binomial logistic regression. A *p*-value of < 0.05 was considered statistically significant. A post hoc power analysis was conducted using G*Power 3.1.9.7 [21].

## Figures and Tables

**Table 1:** Physicians’ backgrounds.

□	Generalists	Pulmonologists	Otolaryngologists
Number	660	220	220
Female	71 (11)	23 (10)	28 (13)
Years of career as a physician, year	27 (19–33)	20 (13–27)	25 (15–33)
Awareness of the recommendation	346 (52)	147 (67)	119 (54)
Number of attendances in training sessions per year, and number	2 (1–5)	3 (2–5)	2 (1–3)
Certified infectious disease specialist	54 (8)	43 (20)	6 (3)
Involvement in management	310 (50)	64 (29)	97 (44)
Examining children on a daily basis	369 (56)	72 (33)	213 (97)

n (%) or median (IQR).

**Table 2:** Fictitious scenarios to determine the intention to prescribe antibiotics.

	Adult/Child	Diagnosis	Antibiotics
1	Adult	Narrow-defined common cold	Not recommended
2	Adult	Mild acute rhinosinusitis	Not recommended
3	Adult	Moderate or severe acute rhinosinusitis	Recommended
4	Adult	Mild acute pharyngitis	Not recommended
5	Adult	Moderate or severe acute pharyngitis	Recommended
6	Adult	Acute bronchitis without chronic lung disease	Not recommended
7	Adult	Acute bronchitis with chronic lung disease	Recommended
8	Child	Narrow-defined common cold	Not recommended
9	Child	Narrow-defined common cold	Not recommended
10	Child	Acute pharyngitis caused by Group A Streptococcus	Recommended

## Data Availability

Data are contained within the article and Supplementary Materials.

## Supplementary Materials

The following supporting information can be downloaded at: https://www.mdpi.com/article/10.3390/antibiotics13080724/s1, Table S1: The most common reasons for the prescription of antibiotics. Table S2: Associations between the physician’s background and improper use of antibiotics before viewing the educational film. Table S3: Associations between the physician’s background and the positive effects of the educational film. Table S4: Questionnaire for the 10 fictional cases before and after viewing the education film.
