# Supplementary material for: Impact of Educational Films on Antibiotic Prescription among Physicians: A Web-Based Survey in Japan
Source: Antibiotics (Basel). 2024 Aug 1;13(8):724. doi: 10.3390/antibiotics13080724 (PMC11350881; doi:10.3390/antibiotics13080724)
Supplement: Supplementary file 1 [file antibiotics-13-00724-s001.zip › antibiotics-3100756-supplementary.pdf]

Supplementary Table S1. The most common reasons for the prescription of antibiotics.

Case	1		2		3		4		5	
Antibiotics	Not recommended		Not recommended		Recommended		Not recommended		Recommended	
	Reasons	%	Reasons	%	Reasons	%	Reasons	%	Reasons	%
1	Redness of the pharynx	55	Patient's desire	59	Facial pain	82	Pus	86	Pus	75
2	Patient's desire	47	Fever	45	Fever	33	Fever	27	Swelling of the ACLN	54
3	Fever	45	Nasal discharge	20	Nasal discharge	32	Sore throat	22	Fever	28
4	Sore throat	34	Absence of sore throat	10	Prolonged course	25	Patient's desire	21	Sore throat	27
5	Cough	13	Absence of cough	5	Patient's desire	17	Cough	4	Patient's desire	18

Supplementary Table S1. continued

Case	6		7		8		9		10	
Antibiotics	Not recommended		Recommended		Not recommended		Not recommended		Recommended	
	Reasons	%	Reasons	%	Reasons	%	Reasons	%	Reasons	%
1	Purulent sputum	88	Purulent sputum	80	Parent's desire	56	Parent's desire	55	Swelling of the ACLN	58
2	Fever	29	Smoking history	35	Fever	43	Fever	52	Redness of the pharynx	55
3	Patient's desire	22	Fever	29	Nasal discharge	23	Cough	24	Sore throat	44
4	Cough	10	Patient's desire	19	Cough	22	Nasal discharge	23	Fever	33
5	No smoking history	2	Cough	13	Child	8	Child	6	Parent's desire	21

Supplementary Table S2. Associations between the physician's background and improper use of antibiotics before viewing the educational film.

Case	1		2		3		4		5	
Antibiotics	Not recommended		Not recommended		Recommended		Not recommended		Recommended	
	OR	P value	OR	P value	OR	P value	OR	P value	OR	P value
Generalist	0.938 (0.737–1.194)	.605	1.019 (0.777–1.337)	.890	2.078 (1.165–3.709)	.013	0.580 (0.441–0.763)	<.001	1.191 (0.817–1.737)	.364
Pulmonologist	0.737 (0.547–0.992)	.044	0.944 (0.676–1.319)	.735	0.728 (0.365–1.454)	.369	1,217 (0.872–1.697)	.248	1.145 (0.737–1.781)	.547
Pharyngologist	1.497 (1.111–2.019)	.008	1.029 (0.739–1.432)	.866	0.397 (0.169–0.933)	.034	1.941 (1.351–2.788)	<.001	0.642 (0.385–1.609)	.088
Female	0.813 (0.557–1.187)	.813	0.954 (0.622–1.463)	.828	1.862 (0.939–3.691)	.075	1.446 (0.927–2.255)	.104	0.720 (0.376–1.378)	.321
Years of career as a physician, year	1.033 (1.021–1.045)	<.001	1.004 (0.991–1.017)	.521	0.976 (0.952–1.000)	.048	1.018 (1.005–1.031)	.006	0.972 (0.955–0.990)	.002
Awareness of the recommendation	0.628 (0.494–0.798)	<.001	0.828 (0.635–1.081)	.165	1.674 (0.979–2.861)	.06	0.824 (0.634–1.071)	.148	1.400 (0.962–2.037)	.079
Number of attendances in training sessions per year, and number	0.989 (0.971–1.007)	.226	0.996 (0.976–1.016)	.671	0.979 (0.929–1.032)	.425	0.987 (0.970–1.005)	.167	1.002 (0.977–1.027)	.881
Certified infectious disease specialist	0.738 (0.493–1.107)	.142	1.312 (0.852–2.022)	.218	0.793 (0.311–2.022)	.628	0.715 (0.469–1.089)	.118	1.724 (1.011–2.942)	.046
Involvement in management	1.463 (1.150–1.859)	.002	1.217 (0.932–1.589)	.149	0.791 (0.470–1.331)	.377	0.985 (0.758–1.280)	.911	1.211 (0.841–1.744)	.305
Examining children on a daily basis	1.391 (1.093–1.771)	.007	1.326 (1.008–1.746)	.044	0.666 (0.401–1.103)	.114	1.099 (0.845–1.430)	.482	1.268 (0.869–1.852)	.219

Supplementary Table S2. continued

Case	6		7		8		9		10	
Antibiotics	Not recommended		Recommended		Not recommended		Not recommended		Recommended	
	OR	P value	OR	P value	OR	P value	OR	P value	OR	P value
General physician	1.053 (0.821–1.351)	.684	1.029 (0.791–1.340)	.830	0.832 (0.578–1.197)	.321	0.884 (0.621–1.258)	.494	0.715 (0.513–0.997)	.048
Pulmonologist	1.114 (0.818–1.515)	.493	0.653 (0.463–0.922)	.015	0.928 (0.516–1.670)	.803	1.128 (0.652–1.952)	.668	1.456 (0.878–2.414)	.145
Pharyngologist	0.834 (0.616–1.127)	.237	1.412 (1.034–1.929)	.03	1.265 (0.866–1.848)	.224	1.086 (0.749–1.576)	.663	1.221 (0.861–1.730)	.262
Female	1.034 (0.698–1.531)	.867	0.825 (0.539–1.265)	.378	0.730 (0.388–1.376)	.331	0.936 (0.525–1.670)	.824	1.034 (0.605–1.768)	.903
Years of experience, year	1.037 (1.024–1.050)	<.001	0.983 (0.971–0.996)	.009	1.008 (0.990–1.025)	.403	1.013 (0.996–1.030)	.146	0.991 (0.975–1.007)	.254
Awareness of the recommendation	0.822 (0.642–1.052)	.120	1.419 (1.090–1.846)	.009	0.822 (0.568–1.190)	.299	0.651 (0.456–0.930)	.018	0.766 (0.546–1.074)	.122
Number of attendances in training sessions per year, and number	0.996 (0.979–1.013)	.650	0.982 (0.960–1.006)	.139	0.994 (0.967–1.022)	.681	0.983 (0.950–1.016)	.304	0.994 (0.970–1.020)	.664
Certified infectious disease specialist	0.850 (0.564–1.280)	.437	0.931 (0.597–1.452)	.753	2.314 (1.350–3.966)	.002	1.148 (0.646–2.041)	.639	0.995 (0.570–1.736)	.986
Involvement in management	1.180 (0.921–1.512)	.190	0.901 (0.693–1.170)	.435	1.146 (0.794–1.654)	.467	1.084 (0.760–1.545)	.656	0.857 (0.615–1.195)	.363
Examining children on a daily basis	1.118 (0.872–1.433)	.378	1.230 (0.943–1.604)	.127	NA		NA		NA	

OR, odds ratio; NA, not applicable

Supplementary Table S3. Associations between the physician's background and the positive effects of the educational film.

Case	1		2		3		4		5	
Antibiotics	Not recommended		Not recommended		Recommended		Not recommended		Recommended	
	OR	P value	OR	P value	OR	P value	OR	P value	OR	P value
General physician	0.965 (0.750–1.240)	.778	1.026 (0.750–1.403)	.873	1.342 (0.569–3.162)	.502	0.831 (0.653–1.058)	.133	0.654 (0.370–1.153)	.142
Pulmonologist	0.810 (0.592–1.109)	.188	1.160 (0.799–1.685)	.435	1.054 (0.389–2.854)	.918	1,110 (0.826–1.492)	.488	1.593 (0.844–3.009)	.151
Pharyngologist	1.292 (0.955–1.748)	.097	0.820 (0.550–1.222)	.329	0.566 (0.167–1.913)	.359	1.189 (0.884–1.598)	.252	1.135 (0.572–2.254)	.718
Female	0.959 (0.646–1.422)	.835	1.183 (0.739–1.892)	.484	1.661 (0.556–4.966)	.364	1.712 (1.167–2.513)	.006	1.316 (0.578–2.998)	.513
Years of experience, year	1.026 (1.013–1.038)	<.001	1.004 (0.989–1.019)	.609	0.983 (0.946–1.023)	.401	1.011 (0.999–1.023)	.061	0.964 (0.938–0.991)	.010
Awareness of the recommendation	0.680 (0.530–0.871)	.002	0.853 (0.627–1.160)	.31	1.605 (0.681–3.781)	.28	0.893 (0.704–1.133)	.351	1.202 (0.674–2.143)	.534
Number of attendances in training sessions per year, and number	0.992 (0.973–1.011)	.399	0.998 (0.975–1.021)	.861	0.994 (0.929–1.063)	.855	0.987 (0.969–1.006)	.177	0.982 (0.928–1.040)	.537
Certified infectious disease specialist	0.650 (0.416–1.016)	.059	1.065 (0.638–1.780)	.809	0.406 (0.054–3.040)	.380	0.946 (0.632–1.416)	.789	1.310 (0.545–3.151)	.547
Involvement in management	1.252 (0.977–1.605)	.076	1.087 (0.799–1.480)	.595	0.261 (0.089–0.768)	.015	0.804 (0.633–1.021)	.074	0.966 (0.543–1.715)	.905
Examining children on a daily basis	1.103 (0.858–1.481)	.446	1.028 (0.752–1.405)	.862	0.676 (0.301–1.519)	.343	0.818 (0.642–1.040)	.102	0.939 (0.528–1.669)	.830

Supplementary Table S3. continued

Case	6		7		8		9		10	
Antibiotics	Not recommended		Recommended		Not recommended		Not recommended		Recommended	
	OR	P value	OR	P value	OR	P value	OR	P value	OR	P value
General physician	1.067 (0.836–1.363)	.601	1.564 (1.045–2.340)	.030	0.995 (0.631–1.569)	.984	0.902 (0.593–1.372)	.630	0.599 (0.348–1.031)	.064
Pulmonologist	1.057 (0.785–1.424)	.714	0.523 (0.298–0.917)	.024	0.562 (0.236–1.340)	.194	0.936 (0.475–1.846)	.849	1.554 (0.729–3.314)	.254
Pharyngologist	0.857 (0.634–1.158)	.315	0.911 (0.564–1.471)	.702	1.241 (0.775–1.986)	.368	1.153 (0.744–1.788)	.524	1.404 (0.807–2.443)	.229
Female	1.230 (0.843–1.795)	.283	1.471 (0.856–2.528)	.162	0.699 (0.309–1.582)	.39	0.981 (0.496–1.941)	.957	1.389 (0.629–3.070)	.417
Years of experience, year	1.016 (1.004–1.027)	.010	0.977 (0.959–0.995)	.011	1.009 (0.987–1.032)	.410	1.015 (0.995–1.036)	.143	0.991 (0.966–1.017)	.508
Awareness of the recommendation	0.944 (0.742–1.201)	.641	1.377 (0.936–2.027)	.104	0.881 (0.555–1.396)	.589	0.702 (0.460–1.070)	.100	0.778 (0.451–1.342)	.367
Number of attendances in training sessions per year, and number	1.009 (0.992–1.027)	.301	0.959 (0.916–1.004)	.075	1.000 (0.969–1.032)	.987	0.983 (0.944–1.024)	.421	1.007 (0.978–1.038)	.630
Certified infectious disease specialist	1.059 (0.706–1.588)	.783	0.822 (0.416–1.622)	.571	1.777 (0.922–3.425)	.086	0.842 (0.403–1.761)	.648	0.478 (0.145–1.575)	.225
Involvement in management	1.025 (0.805–1.305)	.843	0.746 (0.506–1.100)	.139	1.247 (0.786–1.978)	.348	1.145 (0.749–1.749)	.531	0.758 (0.442–1.300)	.315
Examining children on a daily basis	1.027 (0.805–1.311)	.828	0.995 (0.679–1.458)	.980	NA		NA		NA	

OR, odds ratio, NA, not applicable

Supplementary Table S4. Questionnaire for the 10 fictional cases before and after viewing the education film.

Case	Scenario	Questionnaire
1	A male in his 50s visited for nasal discharge, sore throat, cough, and fever (38°C) persistent for 3 days. Redness of the pharynx is observed. He desired antibiotics.	□ Prescribe antibiotics. □ Not prescribe antibiotics.<br>-If you choose "prescribe antibiotics," please select the reasons.<br>□ Fever; □ Nasal discharge; □ Sore throat; □ Cough; □ Redness of the pharynx; □ Patient's desire□ Profit for clinic or hospital<br>-If you choose "not prescribe antibiotics," please select the reasons.<br>□ Suspect of viral infections; □ Not recommended by the guidelines; □ Concern of adverse effects; □ Suspect of bacterial infection but mild; □ No knowledge of appropriate antibiotics type; □ Long duration of symptoms; □ Not older patient
2	A male in his 50s presented with yellow nasal discharge and fever (38°C) for 3 days. He does not complain of a sore throat or cough. He desired antibiotics.	□ Prescribe antibiotics. □ Not prescribe antibiotics.<br>-If you choose "prescribe antibiotics", please select the reasons.<br>□ Fever; □ Nasal discharge; □ No sore throat; □ No cough; □ Patient's desire□ Profit for clinic or hospital<br>-If you choose "not prescribe antibiotics," please select the reasons.<br>□ Suspect of viral infections; □ Not recommended by the guidelines; □ Concern of adverse effects; □ Suspect of bacterial infection but mild; □ No knowledge of appropriate antibiotics type; □ Short duration of symptoms; □ Not older patient
3	A male in his 50s presented with yellow nasal discharge and fever (38°C) for 10 days. He complained of severe facial pain with no sore throat or cough. He desired antibiotics.	□ Prescribe antibiotics. □ Not prescribe antibiotics.<br>-If you choose "prescribe antibiotics," please select the reasons.<br>□ Fever; □ Nasal discharge; □ Facial pain; □ No sore throat; □ No cough; □ Long duration of symptoms; □ Patient's desire□ Profit for clinic or hospital<br>-If you choose "not prescribe antibiotics," please select the reasons.<br>□ Suspect of viral infections; □ Not recommended by the guidelines; □ Concern of adverse effects; □ Suspect of bacterial infection but mild; □ No knowledge of appropriate antibiotics type; □ Short duration of symptoms; □ Not older patient
4	A male in his 50s visited the hospital with a	□ Prescribe antibiotics. □ Not prescribe antibiotics.

	persistent sore throat, cough, and fever (37°C) for 5 days. He did not complain of nasal discharge. A white lesion of his laryngopharynx is observed. The anterior cervical lymph nodes are not swollen. He desired antibiotics.	-If you choose “prescribe antibiotics,” please select the reasons.<br>□ Fever; □ Sore throat; □ Cough; □ No nasal discharge; □ White lesion of pharynx; □ No anterior cervical lymph node swollen; □ Patient’s desire □; Profit for clinic or hospital<br>-If you choose “not prescribe antibiotics,” please select the reasons.<br>□ Suspect of viral infections; □ Not recommended by the guidelines; □ Concern of adverse effects; □ Suspect of bacterial infection but mild; □ No knowledge of appropriate antibiotics type; □ Short duration of symptoms; □ Not older patient
5	A male in his 50s visited for sore throat, appetite loss, and fever (37°C) persistent for 5 days. He did not complain of nasal discharge or cough. A white lesion of his laryngopharynx is observed. The anterior cervical lymph nodes are swollen. He desired antibiotics.	□ Prescribe antibiotics. □ Not prescribe antibiotics.<br>-If you choose “prescribe antibiotics,” please select the reasons.<br>□ Fever; □ Sore throat; □ No cough; □ No nasal discharge; □ White lesion of pharynx; □ Anterior cervical lymph node swollen; □ Patient’s desire□; Profit for clinic or hospital<br>-If you choose “not prescribe antibiotics,” please select the reasons.<br>□ Suspect of viral infections; □ Not recommended by the guidelines; □ Concern of adverse effects; □ Suspect of bacterial infection but mild; □ No knowledge of appropriate antibiotics type; □ Short duration of symptoms; □ Not older patient
6	A male in his 50s without a history of smoking visited the hospital with a persistent cough, purulent sputum, and fever (37°C) for 5 days. He did not complain of nasal discharge or sore throat. Abnormal lung involvement on chest X-ray was not observed. He desired antibiotics.	□ Prescribe antibiotics. □ Not prescribe antibiotics.<br>-If you choose “prescribe antibiotics,” please select the reasons.<br>□ No smoking history; □ Fever; □ Cough; □ Purulent sputum; □ No nasal discharge; □ No sore throat; □ No abnormal features on chest X-ray; □ Patient’s desire; □ Profit for clinic or hospital<br>-If you choose “not prescribe antibiotics,” please select the reasons.<br>□ Suspect of viral infections; □ Not recommended by the guidelines; □ Concern of adverse effects; □ Suspect of bacterial infection but mild; □ No knowledge of appropriate antibiotics type; □ Short duration of symptoms; □ Not older patient
7	A male in his 50s with a history of heavy smoking presented at the hospital with a persistent cough, purulent sputum, and fever	□ Prescribe antibiotics. □ Not prescribe antibiotics.<br>-If you choose “prescribe antibiotics,” please select the reasons.<br>□ Smoking history; □ Fever; □ Cough; □ Purulent sputum; □ No nasal discharge; □ No sore throat; □ No abnormal

	(37℃) for 5 days. He did not complain of nasal discharge or sore throat. Abnormal lung involvement on chest X-ray was not observed. He desires antibiotics.	features on chest X-ray; □ Patient's desire□ Profit for clinic or hospital<br>-If you choose "not prescribe antibiotics," please select the reasons.<br>□ Suspect of viral infections; □ Not recommended by the guidelines; □ Concern of adverse effects; □ Suspect of bacterial infection but mild; □ No knowledge of appropriate antibiotics type; □ Short duration of symptoms; □ Not older patient
8	A 2-year-old preschool child visited the hospital with nasal discharge and a persistent cough (past 3 days) and fever (38℃; past one day). The accompanying parents desired antibiotic treatment.	□ Prescribe antibiotics. □ Not prescribe antibiotics.<br>-If you choose "prescribe antibiotics," please select the reasons.<br>□ Fever; □ Cough; □ Nasal discharge; □ Being child; □ Parent's desire; □ Profit for clinic or hospital<br>-If you choose "not prescribe antibiotics," please select the reasons.<br>□ Suspect of viral infections; □ Not recommended by the guidelines; □ Concern of adverse effects; □ Suspect of bacterial infection but mild; □ No knowledge of appropriate antibiotics type; □ Short duration of symptoms; □ Profit for clinic or hospital
9	A 10-year-old child visited the hospital with nasal discharge and cough for the past 3 days and fever (38°C) for the past one day. The accompanying parents desired antibiotic treatment.	□ Prescribe antibiotics. □ Not prescribe antibiotics.<br>-If you choose "prescribe antibiotics," please select the reasons.<br>□ Fever; □ Cough; □ Nasal discharge; □ Being child; □ Parent's desire; □ Profit for clinic or hospital<br>-If you choose "not prescribe antibiotics," please select the reasons.<br>□ Suspect of viral infections; □ Not recommended by the guidelines; □ Concern of adverse effects; □ Suspect of bacterial infection but mild; □ No knowledge of appropriate antibiotics type; □ Short duration of symptoms; □ Profit for clinic or hospital
10	A 5-year-old child visited us for a severe sore throat, fever (38°C), and stomach ache for one day. Nasal discharge and cough were not present. A white lesion was seen on the laryngopharynx, and the anterior cervical lymph nodes were swollen. The accompanying parents desired antibiotic	□ Prescribe antibiotics. □ Not prescribe antibiotics.<br>-If you choose "prescribe antibiotics", please select the reasons.<br>□ Fever; □ Sore throat; □ No cough; □ No nasal discharge; □ Redness of pharynx;□ Anterior cervical lymph node swollen; □ Parent's desire; □ Profit for clinic or hospital<br>-If you choose "not prescribe antibiotics," please select the reasons.<br>□ Suspect of viral infections; □ Not recommended by the guidelines; □ Concern of adverse effects; □ Suspect of bacterial infection but mild; □ No knowledge of appropriate antibiotics type; □ Short duration of symptoms; □ Not

	treatment.	older patient; □ Profit for clinic or hospital
